# Physiochemical, Optical and Biological Activity of Chitosan-Chromone Derivative for Biomedical Applications

**DOI:** 10.3390/ijms13056102

**Published:** 2012-05-18

**Authors:** Santosh Kumar, Joonseok Koh

**Affiliations:** Department of Textile Engineering, Konkuk University, Seoul 143-701, Korea; E-Mail: santoshics@rediffmail.com

**Keywords:** chitosan, chitosan-chromone derivative, characterization, biomaterial

## Abstract

This paper describes the physiochemical, optical and biological activity of chitosan-chromone derivative. The chitosan-chromone derivative gels were prepared by reacting chitosan with chromone-3-carbaldehyde, followed by solvent exchange, filtration and drying by evaporation. The identity of Schiff base was confirmed by UV-Vis absorption spectroscopy and Fourier-transform infrared (FTIR) spectroscopy. The chitosan-chromone derivative was evaluated by X-ray diffraction (XRD), thermogravimetric analysis (TGA), differential scanning calorimetry (DSC), scanning electron microscopy (SEM), photoluminescence (PL) and circular dichroism (CD). The CD spectrum showed the chitosan-chromone derivative had a secondary helical structure. Microbiological screening results demonstrated the chitosan-chromone derivative had antimicrobial activity against *Escherichia coli* bacteria. The chitosan-chromone derivative did not have any adverse effect on the cellular proliferation of mouse embryonic fibroblasts (MEF) and did not lead to cellular toxicity in MEFs. These results suggest that the chitosan-chromone derivative gels may open a new perspective in biomedical applications.

## 1. Introduction

Chitosan is an abundant heteropolysaccharide obtained from deacetylation of chitin, which is a polysaccharide of natural origin and second most abundant to cellulose. Considerable interest to researchers lies in the biocompatible, biodegradable, cytocompatible, hemocompatible and adsorption properties of chitosan, which can be exploited to create unique building blocks with novel function via Schiff base [1–4]. These properties are attracting interest for use in pharmaceutical and biomedical fields, including as antimicrobials, gene delivery, carriers of immobilized enzymes and cells, biosensors, artificial organs, and biodegradable packaging, as well as wound healing and as scaffolds for tissue regeneration [5–16]. Chitosan has a natural ability to bind metal ions. Misra *et al*. have reported a chitosan-grafted copolymer magnetic nanoparticle drug carrier for controlled drug release that responds to the change in external temperature or pH, with characteristics of longer circulation time and reduced side effects [17–19]. The heteropolysaccharide backbone of chitosan is structurally similar to glycosaminoglycans, the major component of the extracellular matrix of the bone [20]. Chitosan is also an osteoconductive [21] and hemostatic material [22,23]. The organic biopolymer containing chromophoric groups, whose optical property make the material much more important from a biotechnological and medicinal application point of view [24–26].

Chromones (4*H*-chromone-4-ones) are heterocyclic compounds with the benzo-γ-pyrone framework. Chromones and their structural analogs are of great interest because of their usefulness as biologically active agents. They have been shown to be tyrosine and protein kinase inhibitors, as well as anticancer [27–30], neuroprotective [31], anti-AIDS [32–34], antimicrobial [35,36], antifungal [37], antioxidant activity [38], interleukin-5 inhibitory activity [39], and antiproliferative agents [40]. Chromones may also have application in cystic fibrosis treatment, as they activate the cystic fibrosis transmembrane conductance regulator. Kumar et al. have reported a chromone analog inhibits TNF-*α* induced expression of cell adhesion molecules on human endothelial cells via blocking NF-κB activation [41]. Chromone derivatives may also be useful for other applications in medicinal chemistry, such as preparation of fluorescence probes, due to the photochemical properties of chromones. M. E. Badawy [42] reported fungicidal activity of the *N*-(methyl-4*H*-chromene-4-one) chitosan against *Pythium debaryanum* and *Fusarium oxysporum*.

Although chromones have great pharmaceutical importance, chitin and chitosan are cheaper biopolymers. As part of our continuing interest in chitosan modification [2,3,24,25,43], we became interested in preparation of chitosan-chromone derivative gels. There is no previous report on the optical properties of chitosan-chromone derivative gel in the literatures. In this paper, we report the physiochemical, optical and biological activity of chitosan-chromone derivative gel for biomedical applications.

## 2. Results and Discussion

**Scheme 1:**
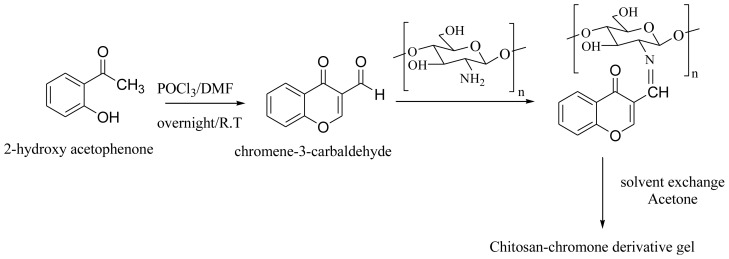
Schematic representation of the preparation of chitosan-chromone derivative gel (Schiff base).

The presence of free reactive amino groups leads to the possibility of forming a Schiff base of chitosan with chromone-3-carbaldehyde. The preparation of chitosan-chromone derivative is shown in Scheme 1. The hydrogel was subject to solvent exchange to remove water and resulting in the xerogel. UV-Vis and FTIR spectroscopy were used to confirm the structure of the Schiff base of chitosan.

### 2.1. FTIR Spectra

**Figure 1:**
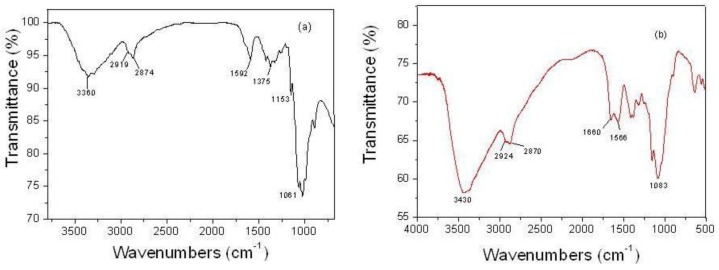
Fourier transform infrared (FT-IR) spectra of pure chitosan (**a**) and chitosan-chromone derivative (**b**).

Figure 1a,b shows the FTIR spectra of chitosan and chitosan-chromone derivative, respectively. The pure chitosan spectrum in Figure 1a shows several characteristic peaks at 3360, 2919, 2874, 1640, 1592, 1375, 1153, 1061 and 893 cm^−1^ [44]. The characteristic peak’s assignment of the chitosan-chromone derivative (Figure 1b) at 1660 cm^−1^ is due to Schiff base (C=N) formed by a cross linking reaction between the amino group and the aldehydic group of chromone-3-carbaldehyde [45,46]. The C=O adsorption peak of secondary hydroxyl groups becomes stronger and moves to 1083 cm^−1^; the intensity of primary alcohol 1034 cm^−1^ due to C=O stretching vibration becomes much smaller than in pure chitosan. 3460 cm^−1^ (O–H group of chitosan), 2924 and 2870 cm^−1^ (C–H stretch), 1566 cm^−1^ and (C=C stretch of aromatic ring) [47], of glucosamine residue. In the chitosan-chromone derivative, the peak at 1592 cm^−1^ disappeared due to the loss of free amine indicating Schiff base amine functionality.

### 2.2. X-ray Diffraction (XRD) Study

**Figure 2:**
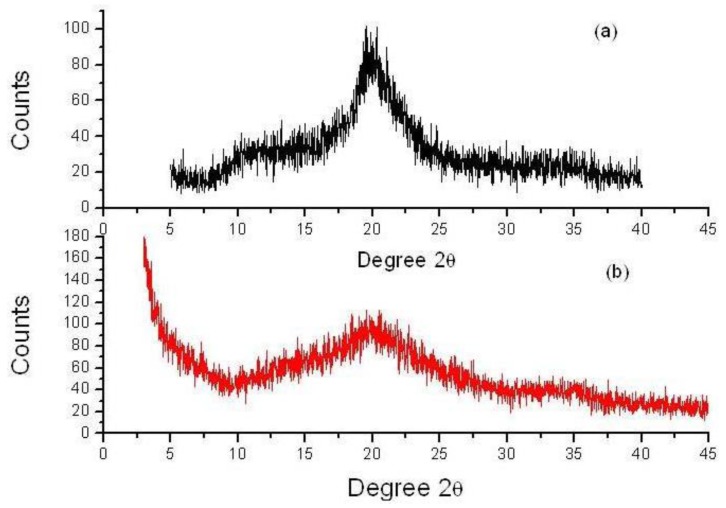
X-ray Diffraction (XRD) pattern of pure chitosan (**a**) and chitosan-chromone derivative (**b**).

X-ray diffraction studies of pure chitosan exhibit very broad peaks at 2*θ* = 10° and 2*θ* = 20° (Figure 2a) [24]. The chitosan-chromone derivative displayed two weak peaks at around 2*θ* of 20° and 35° (Figure 2b). However, the peak observed for chitosan at 2*θ* = 10° disappeared and the very broad peak at 2*θ* = 20° became weak in chitosan-chromone derivative. These results suggest that chitosan has good compatibility, which leads to the formation of a porous xerogel network. The XRD pattern also indicated that the chitosan-chromone derivative displays an amorphous form, which may participate in biomedical applications.

### 2.3. Thermal Analysis (TGA, DSC)

**Figure 3:**
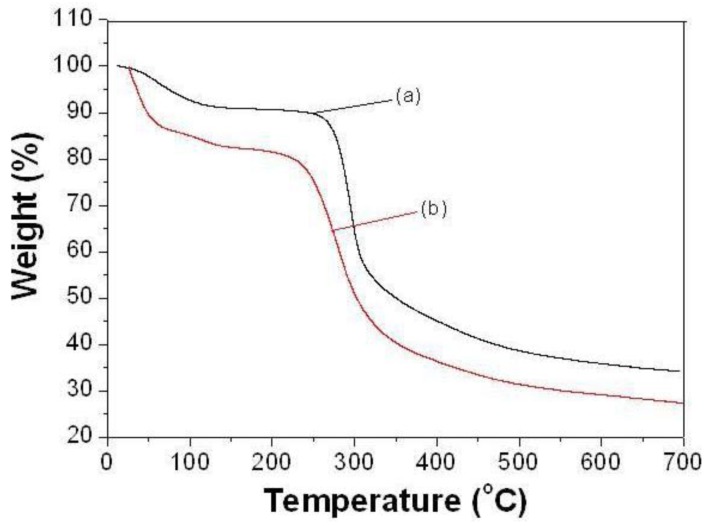
Thermogravimetric analysis (TGA) of pure chitosan (**a**) and chitosan-chromone derivative (**b**).

The TGA thermograms of pure chitosan and chitosan-chromone derivative are shown in Figure 3a,b. The TGA curve of pure chitosan shows that the two stages of weight loss is in the range from 47 to 450 °C, the first occurring in the range of 47–100 °C due to loss of water molecules with a weight loss of about 9%. The primary degradation of pure chitosan started at 247 °C and it was completely degraded at about 450 °C with a weight loss of about 34% [24]. TGA of chitosan-chromone derivative showed two different stages of weight loss (Figure 3b). The first stage of weight loss, starting from 29 to 90 °C, may correspond to the loss of adsorbed water. The second decomposition stage occurs in the range 228–400 °C, due to thermal degradation with a weight loss of about 54%. The results demonstrate the loss of the thermal stability for the chitosan-chromone derivative gel compared to the chitosan.

**Figure 4:**
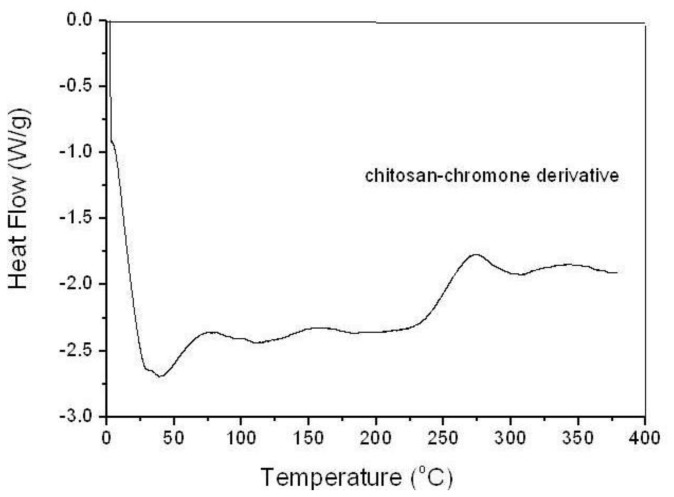
Differential scanning calorimetry (DSC) of chitosan-chromone derivative.

The DSC thermogram of chitosan-chromone derivative is presented in Figure 4. The DSC thermogram of chitosan (not shown) shows two broad endothermic peaks at 92 °C and 212 °C. The first peak may be due to water vapor, while the latter may be attributed to the molecular arrangement of chitosan chains. DSC thermogram of chitosan-chromone derivative (Figure 4) showed characteristic sharp endothermic peaks at 85 °C due to the loss of water molecules. There is one broad exothermic peak at 285 °C corresponding to the thermal decomposition of chitosan-chromone derivative. The results indicated that the structure of chitosan chains have been changed due to the chromone ring and the reduced ability to crystallize.

### 2.4. Scanning Electron Microscopy (SEM)

**Figure 5:**
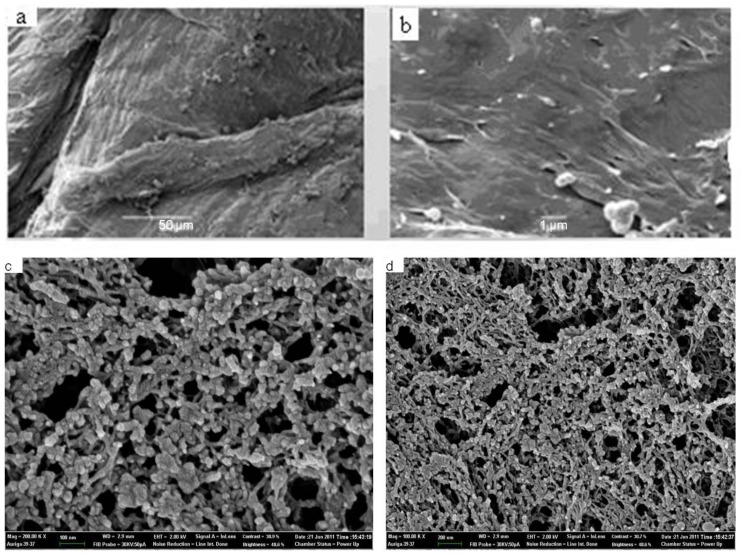
Scanning electron microscopy (SEM) images of pure chitosan (**a**) and (**b**), and chitosan-chromone derivative (**c**) and (**d**).

The SEM images of the pure chitosan (Figure 5a,b) and chitosan-chromone derivative (Figure 5c,d) are shown in Figure 5. The SEM images of pure chitosan exhibited a nonporous, smooth membranous phase consisting of dome shaped orifices, microfibrils and crystallite. The electron micrographs of chitosan-chromone derivative gels (Figure 5c,d) exhibited a porous and chain-like shape. Chitosan-chromone derivative gels also exhibited a cross-section of randomly oriented grains and also gave an image of the upper part of bread slice. The SEM image also confirmed the point that the chitosan-chromone derivative has a near spherical morphology, which may participate into biomedical applications.

### 2.5. Photoluminescence Properties (PL)

**Figure 6:**
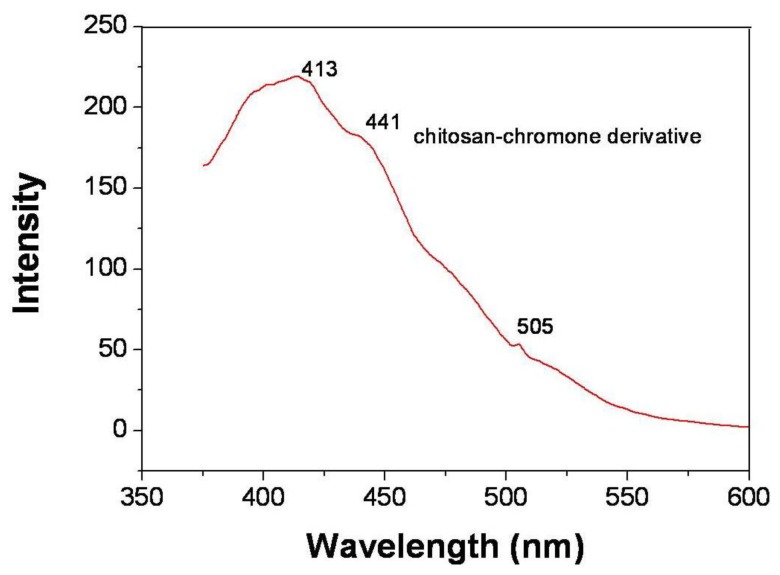
Photoluminescence (PL) spectrum of chitosan-chromone derivative at the excitation wavelength of 320 nm.

Photoluminescence spectra are powerful tools with which to investigate the effect of the chitosan-chromone derivative on optical property. The emission spectra and fluorescent intensity of chitosan-chromone derivative are performed at their own excitation wavelength as shown in Figure 6. The emission spectra of chitosan-chromone derivative (*λ*_em_) peak at 413 nm, 441 nm and 505 nm at excitation wavelength of 320 nm. The chitosan-chromone derivative showed red-shifted emission maximums due to the introduction of side chain and the electronic effect of the substituent on the side chain. This is because the attachment of the conjugate side group to the backbone will enlarge the degree of delocalized π-bond on the electron rich polymer main chain. The emission intensity was greatly influenced by the conjugation length and variation of the substituents. The p-π conjugated side chain can make the emission maximum of chitosan-chromone derivative red shift [24,25]. The shifting wave number was decided by electronic effect.

### 2.6. UV-Vis Absorption Spectrum and Circular Dichroism (CD) Spectrum

**Figure 7:**
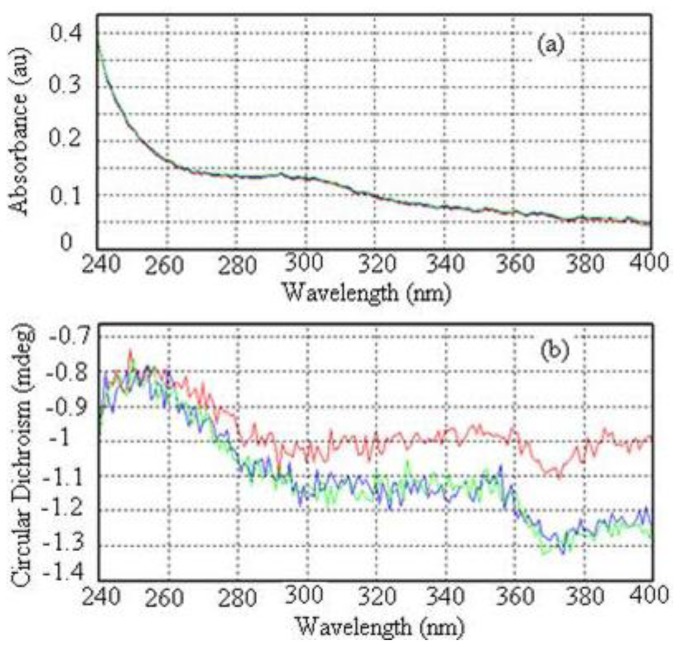
UV (**a**) and Circular dichroism (CD) (**b**) spectra of the chitosan-chromone derivative.

The UV-vis absorption spectrum of the chitosan-chromone derivative is shown in Figure 7a. As shown in Figure 7a, the absorption peak of the chitosan-chromone derivative was 305 nm; this is due to the presence of the chromone heterocyclic ring. Chitosan itself is transparent in the UV-Vis region, and so its structure is hard to characterize by UV-Vis spectroscopic methods. However, we can overcome this natural handicap by borrowing chromophores from an extrinsic molecule.

The investigation of the circular dichroism spectrum of the chitosan-chromone derivative is shown in Figure 7b at different time intervals. The spectrum in Figure 7b shows 0, 1 and 2 times in red, blue and green, respectively. A cotton effect in the CD spectrum in the range *λ* = 360 nm to 380 nm is characteristic for the helical structure of chitosan derivative and originates from the n-π^*^ transition of the helically arranged imine groups in the polymer backbone [48,49]. In the vicinity of absorption bands of the chitosan derivative, which shows positive or negative absorption, curves take on a characteristic shape, and this behavior is known as the Cotton effect. The CD spectrum of the chitosan-chromone derivative, showed no positive Cotton effect at *λ* = 370 nm, but instead a broad negative signal around *λ* = 370 nm with a decreased intensity (Figure 7b). The change in the circular dichroism spectrum could be the result of the prolonged time in solution leading to partial unfolding of the helical organization, in the presence of the chromone ring.

### 2.7. Antimicrobial Study

**Figure 8:**
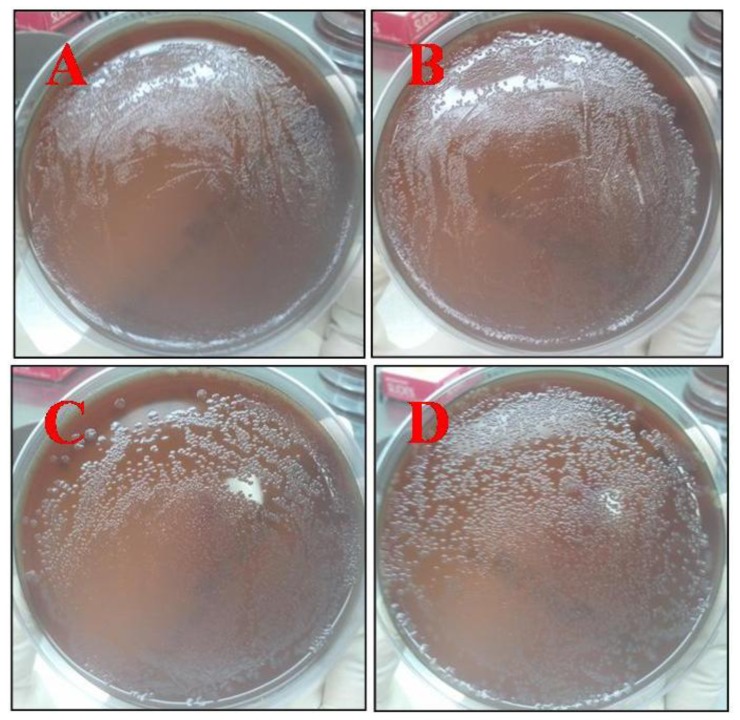
Colony forming units (CFU) of *E. coli* cultured in the presence of chitosan-chromone derivative at a concentration of 0.1 (**A**), 0.01 (**B**), 0.001 (**C**) or 0.0001% (**D**).

The chitosan-chromone derivative had a dose-dependent antimicrobial activity against *E. coli* showing CFUs of less than 2 × 10^8^ when present at a concentration of 0.1% (Figure 8). The most plausible reason for the antimicrobial character of the chitosan-chromone derivative is due to the fact that it has a positively charged amino group which can interacts with the negatively charged microbial cell membranes to cause the leakage of intracellular constituents of the microorganisms, thereby resulting in microbial death [44].

### 2.8. Assay for Cellular Cytotoxicity, Proliferation, and Viability

**Figure 9:**
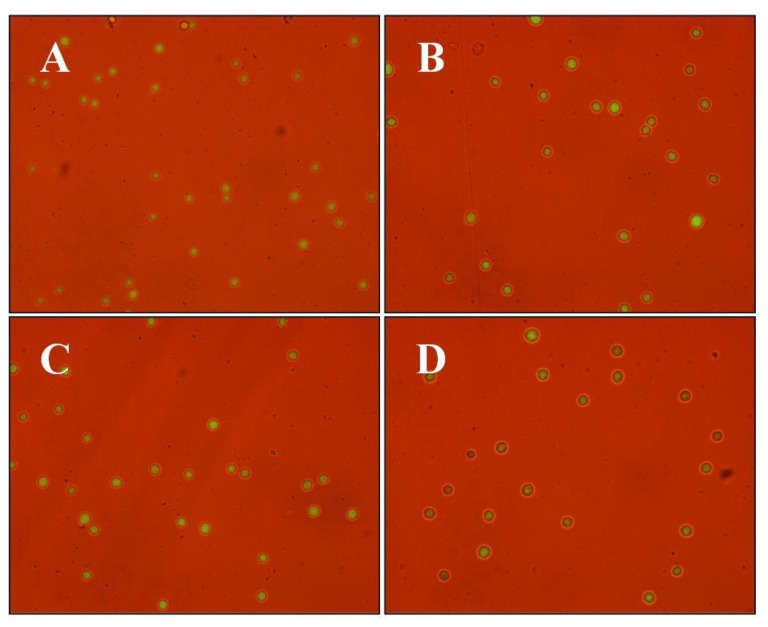
Cellular viability of mouse embryonic fibroblast (MEF) cells cultured in the presence of 0 (**A**), 40 (**B**), 80 (**C**), or 800 μg/mL (**D**) chitosan-chromone derivative. Viability was measure based on esterase enzyme activity and plasma membrane integrity by FDA (3′,6′-diacetyl fluorescein diacetate) assay. Green fluorescence indicates viable cells.

**Figure 10:**
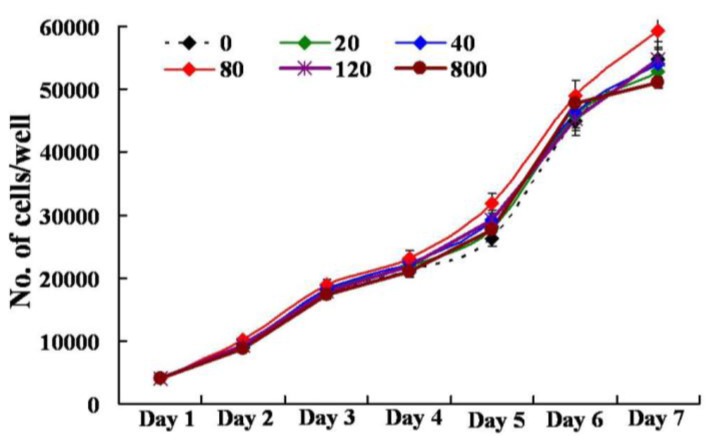
Cellular proliferation of mouse embryonic fibroblast (MEF) cells cultured in the presence of chitosan-chromone derivative at different concentrations as indicated (μg/mL).

The presence of the chitosan-chromone derivative (0, 20, 40, 80, 120, 800 μg/mL) did not had any adverse affect (*p* > 0.05) on the cellular viability of mouse embryonic fibroblast (MEF) cells in culture, as measured by cytoplasmic esterase enzyme activity and plasma membrane integrity (Figure 9 and Table 1). The chitosan-chromone derivative treated cells also had a similar rate of growth as observed with non-treated control cells (Figure 10). The mean population doubling time (PDT) and rate of proliferation per day (*r*) did not differ between non-treated control and chitosan-chromone derivative treated cells for all the tested concentrations (Table 1; *p* > 0.05). These data clearly suggest that the chitosan-chromone derivative was non-cytotoxic and had no adverse effect on the cellular proliferation.

## 3. Experimental Section

### 3.1. Synthesis of 4-oxo-4*H*-Chromene-3-Carbaldehyde

POCl_3_ (6 mol) was added drop wise to 2-hydroxy acetophenone in 50 mL DMF (1 mol) for 15 min at 0 °C. After, the reaction was allowed to stand at room temperature overnight. Reaction mixture was quenched with water and extracted with ethyl acetate. The resulting organic layer was washed with brine, dried over Na_2_SO_4_ and concentrated under reduced pressure to give a pure compound. The yield 82%, a yellow solid, was obtained according to the procedure described elsewhere [50].

### 3.2. Preparation of Chitosan-Chromone Derivative

For the preparation of the chitosan-chromone derivative, 500 mg of chitosan powder was dissolved in 25 mL of 1.5% (*v*/*v*) glacial acetic acid. The mixture was vigorously stirred by a magnet stirrer at room temperature until the polymer was completely dissolved. 100 mg of chromone-3-carbaldehyde in ethanol solution was added drop wise into the chitosan solution and stirred at room temperature for 4 h. The prepared hydrogel solution was then subjected to solvent exchange into acetone (3 × 50 mL) at room temperature. After that, the product was filtered by vacuum. The final product was dried by evaporation at room temperature.

### 3.3. Characterization Methods

Fourier transform infrared (FT-IR) spectra were recorded on a JASCO FT-IR 300E device using KBr. XRD patterns of the samples were recorded on an X-ray diffractometer (D/Max2500VB+/Pc, Rigaku, Japan) with CuKα characteristic radiation (wavelength *λ* = 0.154 nm) at a voltage of 40 kV and a current of 50 mA. The scanning rate was 3°/min and the scanning scope of 2*θ* was from 2° to 45° at room temperature (25 °C). Thermogravimetric analysis (TGA) was carried out in a TA Q 50 system TGA. The samples were scanned from 0 to 800 °C at a heating rate of 10 °C/min under flow of nitrogen. Differential scanning calorimetry (DSC) was performed using DSC Q1000V7.0 A universal V3.6C TA instrument, with heating and cooling rates of 10 °C min^−1^ was used. The surface morphology was analyzed by scanning electron microscopy (SEM) JEOLJSM-6490LA). The photoluminescence (PL) spectra were recorded on a Perkin-Elmer LS55 fluorescence spectrometer. UV-visible absorption spectrum was measured on an Agilent 8453 spectrophotometer (USA). CD spectrum was recorded on a Jasco-J715 spectrometer in 1% acetic acid. The samples were contained in a 1 cm quartz cuvette. All spectra were taken in room temperature.

#### 3.3.1. Antimicrobial Activity Assay

The chitosan-chromone derivative was tested for antimicrobial activity against *Escherichia coli*. Bacteria were grown in Luria-Bertani (LB) broth at 37 °C for 24 h. Fifty microliters of growing culture were then streaked on Blood Agar Plate (BAP) containing the chitosan-chromone derivative in log dilution (0.0001, 0.001, 0.01, and 0.1%) and incubated at 37 °C for 12 h. At the end of incubation, colony forming units (CFU) was measured for each group.

#### 3.3.2. Assay for Cellular Cytotoxicity, Proliferation and Viability

Primary mouse embryonic fibroblast (MEF) cells were isolated from the ICR mouse strain at 15 days *post-coitum* (dpc), and cultured in Dulbecco’s modified Eagle’s medium (DMEM, high glucose formulation; Gibco BRL, Grand Island, NY) supplemented with 10% (*v*/*v*) fetal bovine serum (Hyclone, Logan, UT), MEM nonessential amino acids (Gibco BRL), 50 μM 2-mercaptoethanol, and chitosan-chromone derivative (0, 20, 40, 80, 120, 800 μg/mL) for seven days at 37 °C in a humidified atmosphere of 5% CO_2_ in air. The cells were plated in 24 well plates at an initial seeding density of 2–4 × 10^4^ cells/mL in triplicate and were evaluated for the rate of cellular toxicity and proliferation by counting the total number of cells every 24 h, as we described earlier [44]. The population doubling time was calculated with the equation *Y*_end_ = *Y*_start_ × 2(*t*/*T*), where *T* is the population doubling time, *Y*_start_ is the initial cell count, and *Y*_end_ is the cell count at the end of culture period (*t*). The rate of cell proliferation (*r*) was calculated with the equation *r* = (logNH − logNI)/*T*_2_ − *T*_1_, where *N*_H_ is number of cell harvested, *N*_I_ is number of cells initially seeded, *T*_1_ is the time at seeding (h), and *T*_2_ is the time till harvesting (h). Viability of cells was evaluated based on the esterase enzyme activity and plasma membrane integrity upon FDA (3′ 6′diacetyl fluorescein diacetate) assay as described earlier [44]. Briefly, cells were washed in Dulbecco’s phosphate-buffered saline (DPBS) for 1 min followed by incubation with 2.5 μg/mL FDA stain for 1 min. Stained cells were then washed in PBS to remove the traces of the dye and observed under UV illumination of an epifluorescent microscope fitted with FITC filter set (excitation: 460–490 nm; emission; 515–550 nm; dichromatic: 505 nm). Live cells emitted green fluorescence while dead ones were non-fluorescent. Viability was calculated as the number of green cells/total number of cells ×100. All experiments were repeated three times.

## 4. Conclusions

Chitosan-chromone derivative xerogels have been prepared by converting the chitosan hydrogels by solvent exchange method. The formation of Schiff base (imine bond) was confirmed by FTIR, UV, and XRD. The TGA and DSC study show that the gels were thermally stable. The circular dichroism of the chromone derivative showed a broad negative signal with a decreased intensity and the PL spectrum displayed a red shift. The morphological study of the chitosan derivative has shown it has a porous structure. Microbiological screening demonstrated that the chitosan-chromone derivative has antimicrobial activity. The assays for cell proliferation and viability showed that the chitosan-chromone derivative was non-cytotoxic. Thus, the chitosan-chromone derivative might be a very promising candidate for practical applications in the fields of biomedical, tissue engineering and biosensors.

## Figures and Tables

**Table 1:** Population doubling time (PDT), cell proliferation rate (*r*) and viability of mouse embryonic fibroblast (MEF) cells cultured in the absence or presence of chitosan-chromone derivative at different concentrations.

Concentration (μg/mL)	PDT (h)	*r*/day	Viability (%)
0	21.33 ^a^	0.81 ^a^	97.53 ^a^
20	21.04 ^a^	0.82 ^a^	96.70 ^a^
40	20.48 ^a^	0.85 ^a^	97.67 ^a^
80	18.96 ^a^	0.93 ^a^	96.34 ^a^
120	20.76 ^a^	0.84 ^a^	96.81 ^a^
800	21.94 ^a^	0.78 ^a^	97.75 ^a^

Three replicates were performed. Values with same superscript (*a*) within column did not differ significantly (*p* > 0.05).
